# Mesenchymal stem/stromal cells primed by inflammatory cytokines alleviate psoriasis-like inflammation via the TSG-6-neutrophil axis

**DOI:** 10.1038/s41419-022-05445-w

**Published:** 2022-11-25

**Authors:** Yayun Ding, Pixia Gong, Junjie Jiang, Chao Feng, Yanan Li, Xiao Su, Xiaojing Bai, Chenchang Xu, Chunxiao Liu, Jianxin Yang, Jiankai Fang, Xiaocao Ji, Yongjing Chen, Peishan Li, Lingchuan Guo, Changshun Shao, Yufang Shi

**Affiliations:** The First Affiliated Hospital of Soochow University, State Key Laboratory of Radiation Medicine and Protection, Institutes for Translational Medicine, Suzhou Medical College of Soochow University, Suzhou, Jiangsu 215123 China

**Keywords:** Autoimmunity, Psoriasis

## Abstract

Psoriasis is currently an incurable skin disorder mainly driven by a chronic inflammatory response. We found that subcutaneous application of umbilical cord- derived mesenchymal stem/stromal cells (MSCs) primed by IFN-γ and TNF-α, referred to as MSCs-IT, exhibited remarkable therapeutic efficacy on imiquimod (IMQ)-induced psoriasis-like inflammation in mice. Neutrophil infiltration, a hallmark of psoriasis, was significantly reduced after treatment with MSCs-IT. We further demonstrated that the effects of MSCs-IT were mediated by tumor necrosis factor (TNF) stimulating gene-6 (TSG-6), which was greatly upregulated in MSCs upon IFN-γ and TNF-α stimulation. MSCs transduced with TSG-6 siRNA lost their therapeutic efficacy while recombinant TSG-6 applied alone could also reduce neutrophil infiltration and alleviate the psoriatic lesions. Furthermore, we demonstrated that TSG-6 could inhibit neutrophil recruitment by decreasing the expression of CXCL1, which may be related to the reduced level of STAT1 phosphorylation in the keratinocytes. Thus, blocking neutrophil recruitment by MSCs-IT or TSG-6 has potential for therapeutic application in human psoriasis.

## Introduction

Psoriasis is a chronic relapsing-remitting skin disease without a cure. It occurs at any age, and is most common in the age group 50–69 with prevalence ranging between 0.09% and 11.4% (as in Norway) [1, 2]. Psoriasis is characterized by inflammatory cell infiltration and abnormal proliferation of keratinocytes [3, 4]. The immunomodulatory drugs targeting the IL-23/IL-17 axis have achieved some success in treating psoriasis [5], however, discontinuance of the therapy often leads to recurrences [6]. Although psoriasis is believed to be driven by dysregulated T cells [7], this notion does not fully explain the whole immune characteristics of this disease. The mechanisms underlying dendritic cell activation and the antigen presented for the activation of T cells during the disease process remain elusive. Immune cells other than T cells involved in the pathogenesis of psoriasis are attracting more attention in the recent years [8, 9]. Indeed, massive neutrophil infiltration is a characteristic of the psoriatic plaques [10, 11]. Moreover, IL-17 secreted by neutrophils has been demonstrated to mediate the inflammation associated with psoriasis [12]. Therefore, neutrophils might be an important player in the pathogenesis of psoriasis.

Neutrophils are traditionally regarded as an effector cell population of innate immunity [13]. However, recent studies showed that neutrophils were also linked to acquired immunity and played a crucial role in the pathogenesis of various diseases, including autoimmunity, infections, inflammation, and cancers [14, 15]. Neutrophils infiltrate and accumulate in the dermis of psoriatic patients at the early phase, and then into the epidermis in the chronic phase [9]. Correspondingly, neutrophil chemoattractants, such as leukotriene B4 (LTB4), CXCL1, and CXCL8 are upregulated in the psoriatic lesions [16, 17]. Neutrophils also function by degranulation, reactive oxygen species (ROS) production, and neutrophil extracellular traps (NETs) releasing [18, 19]. It was reported that inflammation was relieved during agranulocytosis but reappeared after restoration of neutrophils in the blood [20, 21]. Therefore, neutrophils may contribute to the development of psoriasis and could be targeted for treatment.

Mesenchymal stem/stromal cells (MSCs) are multipotent progenitor cells that can modulate immune responses [22, 23]. Interestingly, the immunomodulatory property can be enhanced by inflammatory cytokines [24]. MSCs primed by cytokines, such as IFN-γ and TNF-α, referred to as MSCs-IT, secrete various factors with immunomodulatory activities [25]. However, no specific MSCs-derived mediators have been exploited for treating psoriasis. Moreover, it remains to be determined whether inflammatory cytokines confer MSCs with increased efficacy in treating inflammatory diseases such as psoriasis.

In the present study, we aimed to determine whether MSCs-IT possess enhanced efficacy in treating murine psoriasis-like inflammation induced by imiquimod (IMQ). Subcutaneous injection of MSCs-IT was found to reduce inflammatory symptoms more potently than un-primed MSCs. MSCs-IT alleviated murine psoriasis-like inflammation by producing tumor necrosis factor (TNF) stimulating gene-6 (TSG-6), which inhibited neutrophil infiltration. Importantly, application of TSG-6 alone was sufficient to reduce psoriatic inflammation. Thus, MSCs-IT and TSG-6 might be novel therapeutics for psoriasis.

## Results

### MSCs-IT effectively alleviated imiquimod (IMQ) induced psoriasis-like inflammation in mice

**Fig. 1:**
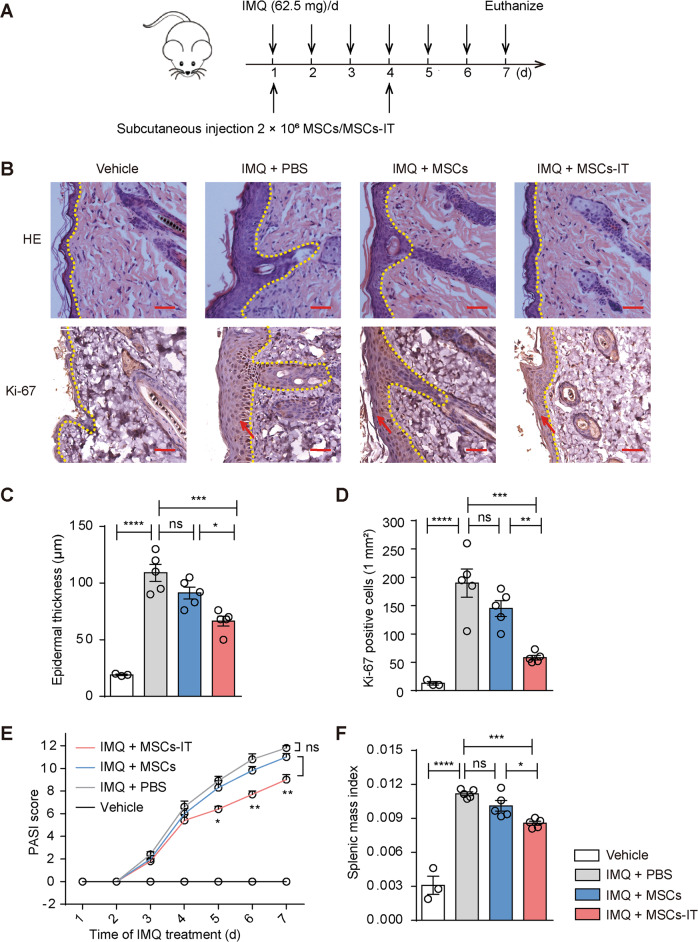
MSCs-IT alleviated imiquimod (IMQ) induced psoriasis-like inflammation of mice. WT mice received subcutaneous injection with 2 × 10^6^ MSCs, MSCs-IT in 150 µL PBS, or 150 µL PBS alone on days 1 and 4 of IMQ application. Mice were euthanized on the 7th day (*n* = 3–5 mice for each group). **A** The experimental scheme. **B** H&E staining of the lesions in each group; the Ki-67 immunostaining of skin in the indicated groups. **C** Measurement of epidermal thickness. **D** The statistics of Ki-67 positive cells. **E** PASI scores of mice in the indicated groups. **F** The splenic index (The spleen weight/The body weight) in each group. Scale bars: 50 µm. Data were shown as means ± SEM, *p < 0.05, **p < 0.01, ***p < 0.001, ****p < 0.0001, ns: not significant.

MSCs activated by IFN-γ and TNF-α possess potent anti-inflammatory and immunosuppressive capabilities and could effectively treat various inflammatory diseases, even systemic infections [23]. Whether MSCs-IT can alleviate skin inflammation such as psoriasis remains to be determined. To address this issue, we applied MSCs derived from the human umbilical cord to treat the IMQ-induced psoriasis-like skin inflammation in mice. IMQ cream was applied daily continuously for six consecutive days. Typical symptoms appeared on the back skin of the mice in the IMQ model group, including scaling, erythema, and thickening. The psoriatic mice were injected with PBS, MSCs, or MSCs-IT subcutaneously on the 1st day and the 4th day after the initial IMQ application. The mice were euthanized on the 7th day (Fig. 1A). Histological examination showed that there was an increase in epidermal thickness in the IMQ model group, which was significantly reduced in the MSC-IT treated group as compared with the other two groups. Likewise, the number of Ki-67 positive cells was also significantly decreased after MSC-IT administration, demonstrating that the proliferation of basal keratinocytes induced by IMQ was reduced after treatment with MSCs-IT (Fig. 1B–D). The severity of inflammation was also evaluated by the Psoriasis Area and Severity Index (PASI) score. The disease symptoms continued to increase along with IMQ application in the IMQ model group. However, the inflammation responses in the MSC-IT group were almost completely relieved as compared with the PBS group. The symptoms of the native MSCs injection group were not relieved (Fig. 1E). The spleen index is another indicator of psoriasis severity. It was also reduced in the MSC-IT group (Fig. 1F). These results demonstrated that MSCs treated with IFN-γ and TNF-α are superior to untreated MSCs in alleviating psoriasis-like lesions in the IMQ model.

### MSC-IT treatment reduced neutrophil accumulation in spleen and psoriatic lesions

**Fig. 2:**
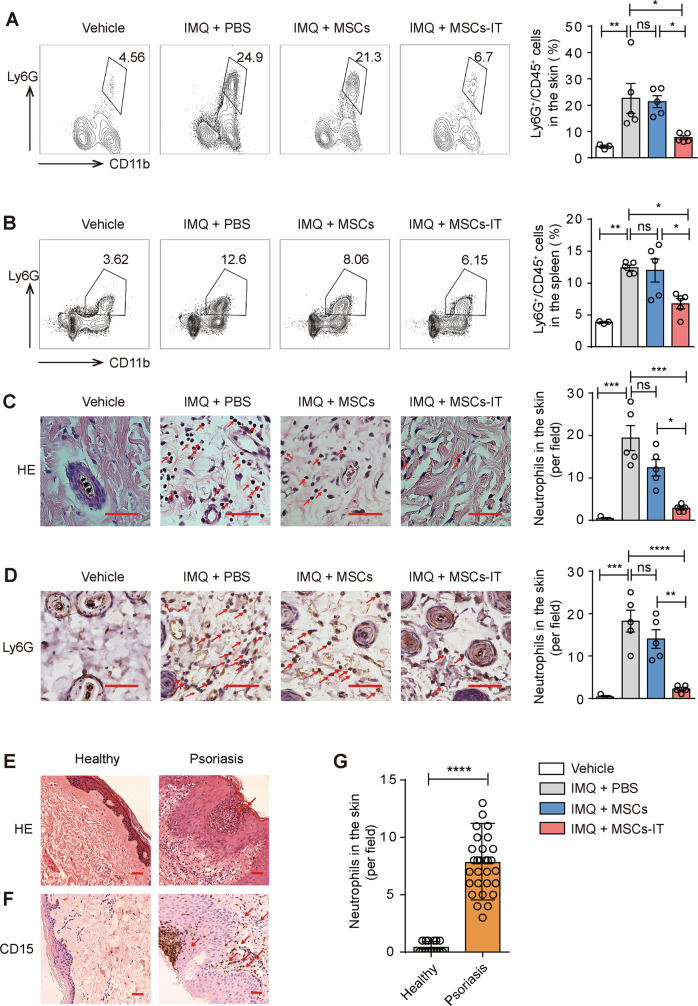
MSCs-IT treatment reduced neutrophil infiltration in psoriatic lesions. Mice were randomly divided into four groups: the vehicle mice, the PBS-treated psoriatic mice, the MSC-treated psoriatic mice, and the MSC-IT-treated psoriatic mice. The mice were euthanized on day 7. The skin and spleen were analyzed by H&E staining, IHC, and flow cytometry. **A, B** Representative images of neutrophils analyzed by flow cytometry of skin and spleen. All frequencies referred to viable cells. Open and filled histogram represented neutrophils in the indicated groups. **C** H&E staining of lesions in the indicated groups. Red arrows highlighted neutrophils. **D** The Ly6G positive cells (neutrophils) of skin were visualized by IHC. Scale bars: 50 µm. Moreover, the specimens of patients were used for H&E staining and IHC analysis. **E** H&E staining of skin in the indicated groups. **F** IHC analysis of neutrophil infiltration in the lesions. Scale bars: 75 µm. **G** Statistics of CD15 positive cells. Data were shown as means ± SEM, *p < 0.05, **p < 0.01, ***p < 0.001, ****p < 0.0001, ns: not significant.

To investigate the mechanisms by which MSCs-IT exert their therapeutic effects on psoriatic inflammation, we examined the immunocytes including T cells, dendritic cells (DC), macrophages, and neutrophils, which are the main contributors to psoriasis, by flow cytometry. The immune cells remained unchanged after treatment except for neutrophils (Fig. 2A and Supplementary Fig. S1). As shown in Fig. 2A, the percentage of neutrophils (CD11b^+^cells and Ly6G^+^cells) was significantly higher in the skin of the IMQ-treated mice, which was dramatically decreased by the MSC-IT treatment. MSCs-IT showed much higher effectiveness than untreated MSCs. Interestingly, MSCs-IT also reduced the percentage of neutrophils in the spleen. IMQ treatment led to increased accumulation of neutrophils in the spleen, which was dramatically reduced by MSCs-IT, even though they were administered locally at the lesion site (Fig. 2B). Again, MSCs-IT were more effective in reducing the neutrophil accumulation in the spleen than naive MSCs. We then employed Ly6G as a marker to examine neutrophils in tissue sections by immunohistochemistry (IHC) and H&E and obtained similar results (Fig. 2C, D). In addition, in tissue specimens of psoriasis obtained from human patients, psoriasis tissue specimens showed classic pathological changes of micro-abscess enriched with neutrophils into the epidermis (Fig. 2E). We further examined neutrophils by IHC staining for CD15, a specific marker of human neutrophils. As expected, neutrophils not only formed Kogoj pustule-like micro-abscess in the epidermis but also infiltrated in the dermis (Fig. 2F, G). Moreover, this result is consistent with a previous study showing that psoriasis is characterized by neutrophil infiltration and the neutrophil reduction is accompanied by the disease regression, and that therapeutic efficacy correlates with neutrophil reduction [20]. Thus, neutrophils probably drive the progression of psoriasis. Together, these results indicate that MSCs-IT could be used to modulate the neutrophil-mediated local and systemic inflammation accompanying psoriatic lesions.

### Neutrophil depletion alleviated psoriatic lesions

**Fig. 3:**
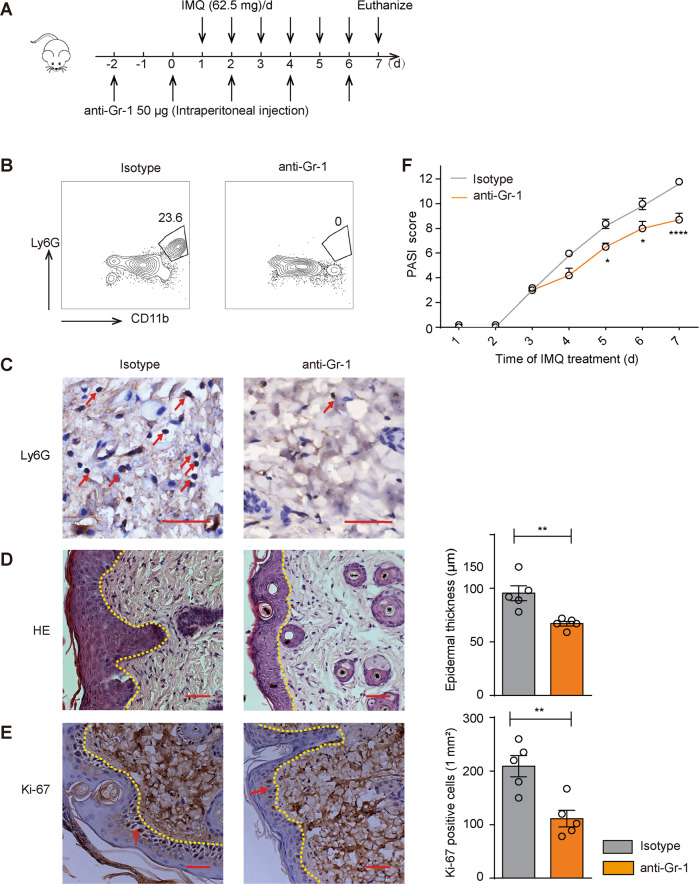
Neutrophil depletion attenuated psoriatic inflammation. The psoriatic mice were randomly assigned into two groups. They were either injected with 50 µg anti-mouse Gr-1 or isotype control antibody (*n* = 5 mice for each group). The anti-mouse Gr-1 was injected intraperitoneally every other day from days −2 to 7. Skin samples on day 7 were for H&E staining and flow cytometry. **A** The experimental scheme. **B** Flow cytometry analysis of neutrophils in the indicated groups. **C** The Ly6G immunostaining of the skin; enumeration of Ly6G positive cells. **D** H&E staining of the skin in the indicated groups; measurement of epidermal thickness. **E** The Ki-67 immunostaining of the skin in both groups; enumeration of Ki-67 positive cells. **F** The PASI scores were monitored daily in the indicated groups during the entire experiment. Data were shown as means ± SEM. Scale bars: 50 µm. *p < 0.05, **p < 0.01, ****p < 0.0001.

Next, we sought to demonstrate the contribution of neutrophils in IMQ model by clearing neutrophils with anti-Gr-1, which depletes neutrophils by complement-dependent pathways [26]. The psoriatic mice were injected anti-Gr-1 intraperitoneally every other day from days -2 to 7 (Fig. 3A). Flow cytometry and Ly6G immunostaining were performed to confirm the successful neutrophil depletion by treatment of anti-Gr-1 (Fig. 3B, C). The symptoms of epidermal thickness and number of Ki-67-positive cells were attenuated (Fig. 3D, E). We assessed the disease severity by PASI score, it revealed that neutrophils depletion could relieve the symptoms of skin inflammation compared with the control group treated with IgG (Fig. 3F). These results suggested that the neutrophils played a critical role in the pathogenesis in the IMQ-induced psoriasis mouse model.

### The therapeutic effects of MSCs-IT on murine psoriasis-like inflammation depended on TSG-6

**Fig. 4:**
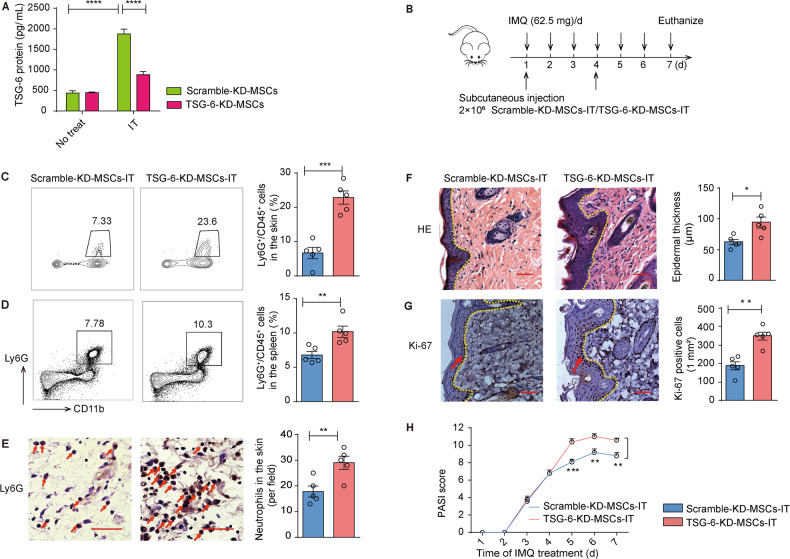
MSCs-IT required TSG-6 to ameliorate psoriatic inflammation. The psoriatic mice were randomly injected subcutaneously with 2 × 10^6^ scramble-KD-MSCs-IT and TSG-6-KD-MSCs-IT suspended in 150 µL PBS on days 1 and 4 (*n* = 5 mice for each group). **A** The concentration of TSG-6 secreted by scramble-KD-MSCs and TSG-6-KD-MSCs with or without cytokine pretreatment for 48 h. **B** The experimental scheme. **C**, **D** TSG-6-KD-MSCs-IT impaired the inhibition of neutrophil infiltration; enumeration of neutrophil frequency in skin and spleen. **E** The Ly6G immunostaining of the skin in both groups; enumeration of Ly6G positive cells. **F** H&E staining of mice in the indicated groups; measurement of epidermal thickness. **G** The Ki-67 immunostaining of the skin in both groups. **H** The PASI scores of psoriatic mice in the indicated groups at all time points. Scale bars: 50 µm. Data were shown as means ± SEM, *p < 0.05, **p < 0.01, ***p < 0.001, ****p < 0.0001.

Various factors have been proven to mediate MSCs-based immunosuppression [27]. To determine the possible effectors of MSCs-IT, we examined the mRNA levels of various anti-inflammatory factors produced by MSCs-IT, including *IDO*, *COX-2*, and *TSG-6* (primers are listed in Supplementary Table. 1). Their expression was greatly increased after treatment with the cytokines (Supplementary Fig. S2). TSG-6 KO mice were shown to have higher neutrophil infiltration in the lung injury model, indicating that TSG-6 might play a critical role in neutrophil recruitment and/or settlement [22]. Thus, we investigated whether TSG-6 mediated the inhibitory effects on neutrophil infiltration by MSCs-IT. We knocked down TSG-6 with siRNA and measured the protein level of TSG-6 in the supernatant by ELISA (Fig. 4A). Then, we treated the psoriatic mice with scramble-KD-MSCs-IT and TSG-6-KD-MSCs-IT, respectively (Fig. 4B). We found TSG-6-KD-MSCs-IT lost their ability to inhibit neutrophil infiltration in skin lesions and the accumulation of neutrophils in spleen (Fig. 4C–E). Furthermore, H&E staining, IHC staining with Ly6G and Ki-67 antibodies, and the PASI score all revealed that the anti-inflammatory effects of MSCs-IT were significantly attenuated by TSG-6-KD-MSCs-IT (Fig. 4F–H). Collectively, our results indicated that TSG-6 secreted by MSCs could alleviate inflammation by restraining neutrophil infiltration in the psoriasis mouse model.

### Recombinant TSG-6 ameliorated murine psoriasis-like inflammation

**Fig. 5:**
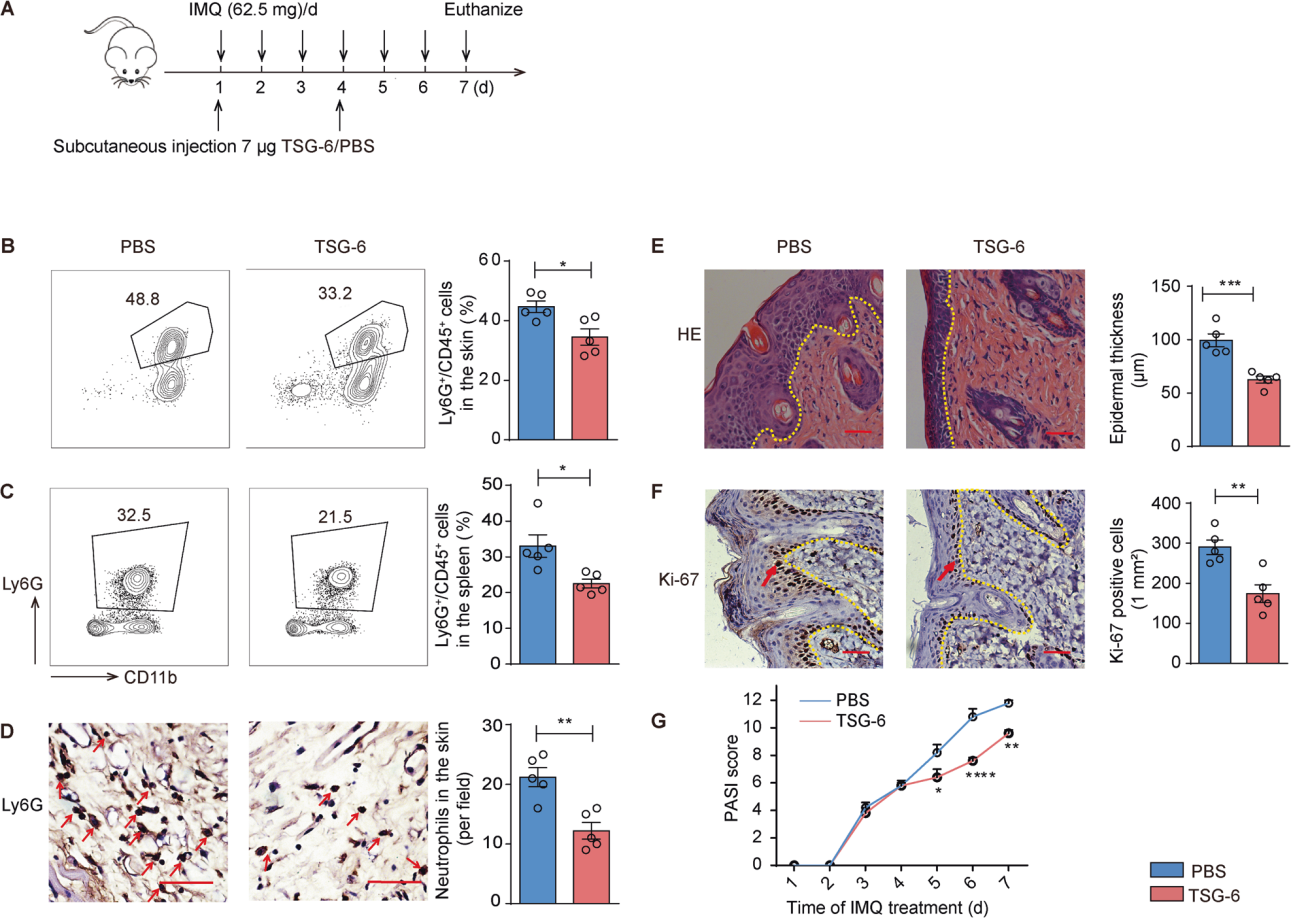
Exogenous TSG-6 ameliorated murine psoriasis-like inflammation. The psoriatic mice were injected subcutaneously with 7 µg rhTSG-6 dissolved with 150 µL PBS or 150 µL PBS alone on days 1 and 4 (*n* = 5 mice for each group). **A** The experimental scheme. **B**, **C** rhTSG-6 impaired neutrophil accumulation in spleen and skin of IMQ-induced psoriatic mice; enumeration of neutrophil frequency in skin and spleen. **D** The Ly6G immunostaining of skin in both groups; enumeration of Ly6G positive cells. **E** H&E staining of lesions; measurement of epidermal thickness. **F** The Ki-67 immunostaining of skin in both groups; enumeration of Ki-67 positive cells. **G** PASI scores of psoriatic mice in the indicated groups at all time points. Scale bars: 50 µm. Data were shown as means ± SEM, *p < 0.05, **p < 0.01, ***p < 0.001, ****p < 0.0001.

To further confirm the role of TSG-6 as an effector of MSCs-IT in alleviating psoriatic inflammation, we applied recombinant human TSG-6 (rhTSG-6) subcutaneously to psoriatic mice. Based on the dosage range of rhTSG-6 injection reported in the previous study [28, 29], rhTSG-6 was injected every three days at the dose of 7 µg per mouse (Fig. 5A). As expected, there were fewer neutrophils that infiltrated in the lesions in the TSG-6 treatment group than in the PBS group (Fig. 5B–D). Furthermore, rhTSG-6 improved the PASI score, reduced the thickness of epidermis and inhibited the proliferation of keratinocytes in psoriatic lesions (Fig. 5E–G). Thus, exogenous rhTSG-6 alone can alleviate psoriatic inflammation.

### MSCs-IT reduced neutrophil infiltration via TSG-6

**Fig. 6:**
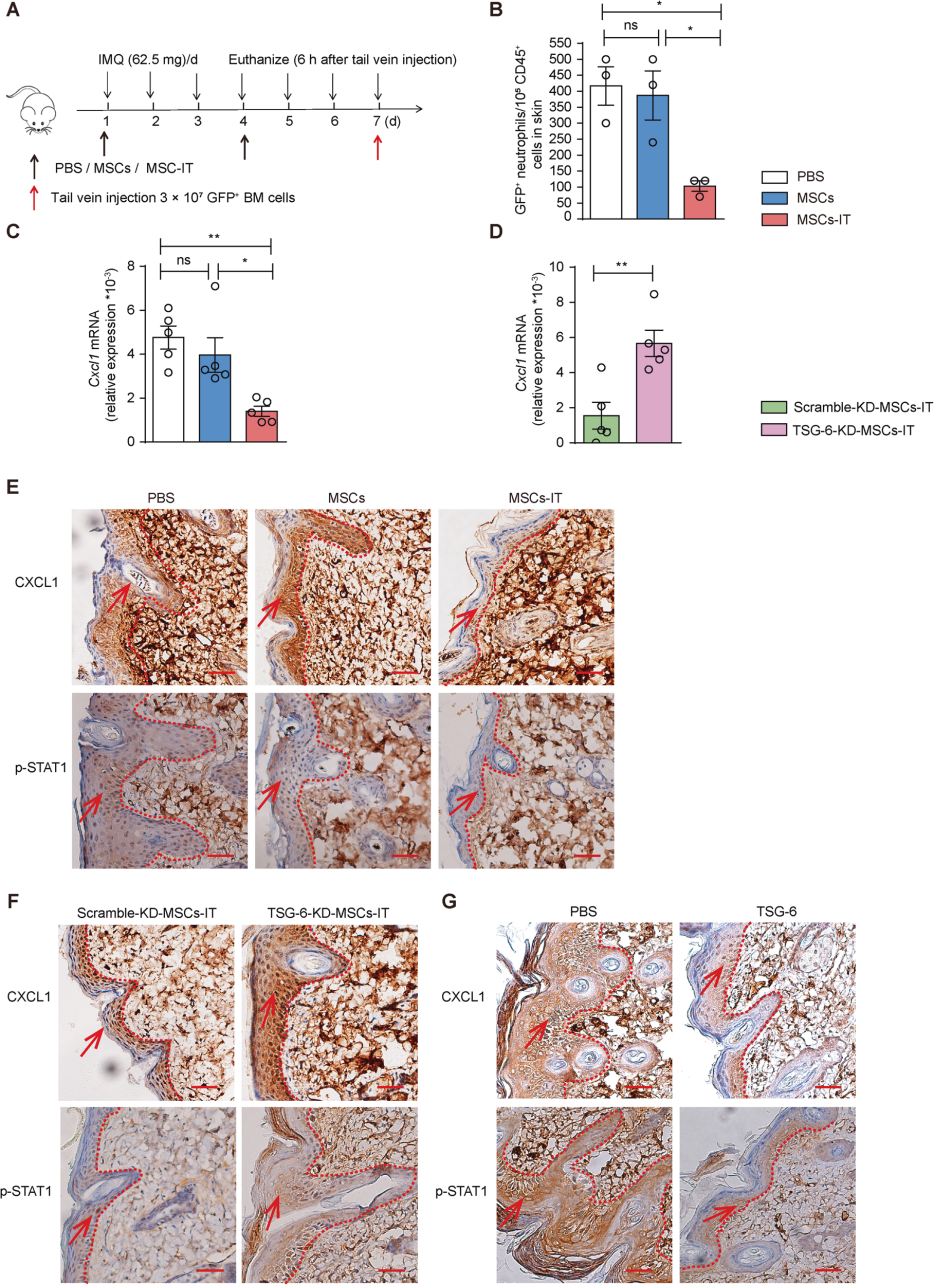
MSCs-IT downregulated CXCL1 via TSG-6. Bone marrow cells from GFP mice were infused into mice on day 7 of MSC treatment. Flow cytometry was used to analyze the proportion of GFP-positive neutrophils in the lesions 6 h later (*n* = 3 mice for each group). **A** The experimental scheme. **B** Enumeration of GFP positive neutrophils in the indicated groups, including IMQ + PBS group, IMQ + MSC group, and IMQ + MSC-IT group. In addition, we further examined the CXCL1 expression (*n* = 5 mice for each group). **C** The relative expression of *Cxcl1* in the indicated groups. **D** The relative expression of *Cxcl1* in the scramble-KD-MSC-IT group and TSG-6-KD-MSC-IT group. **E**–**G** The CXCL1 and p-STAT1 immunostaining of skin in the indicated groups. Scale bars: 50 µm. Data were shown as means ± SEM, *p < 0.05, **p < 0.01, ns: not significant.

Considering that TSG-6 is indispensable in inhibiting neutrophil infiltration of MSCs-IT, we next sought to investigate the mechanisms by which TSG-6 suppressed the neutrophil infiltration. We injected the bone marrow cells obtained from green fluorescent protein (GFP) mice into psoriatic mice and excised the skin lesions 6 h later (Fig. 6A). We found that the number of GFP positive neutrophils in the MSC-IT treatment group was decreased significantly relative to the naive MSC treatment group (Fig. 6B), suggesting that MSCs-IT might decrease the chemotaxis of neutrophils. Examination of the expression of CXCL1, the main chemotactic factor of neutrophils, revealed that the mRNA level of *Cxcl1* was decreased significantly after treatment by MSCs-IT (Fig. 6C). Next, to explore whether this inhibitory effect on neutrophil chemotaxis by MSCs-IT was also mediated by TSG-6, we examined the expression of *Cxcl1* after administration of TSG-6-KD-MSCs-IT. The results showed that the inhibitory effect on chemokine expression was significantly attenuated in the TSG-6-KD-MSC-IT treatment group (Fig. 6D). Furthermore, we confirmed the change of CXCL1 at protein level by employing IHC (Fig. 6E, F). CXCL1 was mainly expressed in keratinocytes [30] and was regulated by p-STAT1 [31, 32]. We, therefore, examined the changes of STAT1 phosphorylation after MSC-IT treatment (Fig. 6E). We found that STAT1 phosphorylation in the keratinocytes was reduced after MSC-IT treatment. Moreover, the inhibitory effect on the phosphorylation of STAT1 was significantly attenuated in the TSG-6-KD-MSC-IT treatment group (Fig. 6F). The downregulation of p-STAT1 and CXCL1 was also observed in rhTSG-6 treated mice (Fig. 6G). Thus, TSG-6 may down-regulate the expression of CXCL1 by inhibiting the phosphorylation of STAT1 in the keratinocytes. Together, the results showed that the neutrophil infiltration in psoriatic lesions could be inhibited by MSCs-IT via TSG-6/CXCL1 axis.

## Discussion

Psoriasis is a chronic recurrent disease without a cure. Although some new biological agents have been approved for psoriasis treatment [33, 34], their therapeutic effects vary greatly between patients, and the disease relapses easily after discontinuation of the drugs [35]. The present study was designed to explore the potential utility of cytokine-primed MSCs in the treatment of murine psoriasis-like inflammation. We observed that MSCs-IT potently reduced the progression of psoriasis by regulating neutrophil-mediated inflammation. Moreover, we demonstrated that TSG-6 secreted by MSCs-IT could inhibit neutrophil recruitment by decreasing the expression of CXCL1, which may be related to the reduced level of p-STAT1. These results suggest that MSCs-IT have the potential for clinical application in the treatment of human psoriasis.

The immunomodulatory ability of MSCs is not innate but is licensed and dictated by the inflammation types and intensity [36]. In fact, different inflammatory environment can lead to markedly different response to MSC treatment. It was shown that graft-versus-host disease (GvHD) can be treated successfully by MSC injection when inflammation was in progress but was less effective before inflammation has begun [37]. Likewise, the therapeutic effect on experimental autoimmune encephalomyelitis was diminished during disease remission [38]. Cytokine-educated MSCs also promote tissue repair. It was shown that MSCs stimulated with IFN-γ and TNF-α produced a large amount of VEGFC to promote angiogenesis and accelerate skin wound healing [39]. In accordance with these studies, we showed that MSCs-IT possesses much more effectiveness than the naive MSCs in treating murine psoriasis-like inflammation, which was in contrast to the previous studies that employed un-primed MSCs [40]. It should be noted that MSCs can be empowered by other means to acquire increased therapeutic efficacy. For example, enhanced therapeutic efficacy on psoriasis mouse model was achieved when MSCs over-expressed SOD3 [41].

We found that TSG-6 was the key molecule to treat murine psoriasis-like inflammation. TSG-6, a 30-kDa hyaluronan-binding protein, is always expressed at a low level constitutively [42]. However, it is secreted by MSCs, fibroblasts, and activated macrophages in response to proinflammatory mediators, such as IL-1β and TNF-α, and plays a critical role in modulating inflammation [43]. It was shown that TSG-6 ameliorated colitis by inhibiting neutrophil infiltration [44]. Interestingly, TSG-6 was also greatly upregulated in muscle stem cells in response to IFN-γ and TNF-α stimulation and mediated their potently therapeutic efficiency in ulcerative colitis [45]. In accordance with these findings, we found that MSCs-IT relieved murine psoriasis-like inflammation by inhibiting the neutrophil infiltration in a manner dependent on TSG-6 production. Our most definitive proof of the key roles of neutrophil infiltration inhibition is that TSG-6-KD-MSCs-IT lack immunosuppressive capability to treat murine psoriasis-like inflammation. Thus, TSG-6 plays a central role in relieving the inflammation of psoriasis mouse model. RhTSG-6 may be an alternate for MSCs-IT to treat murine psoriasis-like inflammation. Also, the other cells overexpressed TSG-6 may also play a central role in therapies for psoriatic mice.

The main mechanisms by which TSG-6 exerts anti-inflammatory effects are as follows: (1) It binds to fragments of hyaluronan and thereby decreases the inflammation [46], (2) It increases the activity of inter-α-inhibitor and then decreases the inflammation [47], (3) It inhibits neutrophil migration to the inflammatory sites mainly by inhibiting Chemokine/Glycosaminoglycan Interactions [48]. The current study showed that MSCs-IT significantly inhibited the CXCL1 expression, whereas TSG-6-KD-MSCs-IT were greatly compromised in that capacity compared with scramble-KD-MSCs-IT. Furthermore, CXCL1 was mainly expressed in keratinocytes and was regulated by p-STAT1. We found that TSG-6 could also reduce the level of STAT1 phosphorylation. Thus, TSG-6 might reduce neutrophil infiltration by inhibiting STAT1 phosphorylation, though a direct link between STAT1 phosphorylation and CXCL1 in the keratinocyte remains to be demonstrated. Additionally, our study does not exclude the possibility that MSCs-IT produces additional effector molecules in addition to TSG-6.

Neutrophils, the most abundant innate immune cells, are related to autoimmune diseases [49]. The neutrophil infiltration in skin lesions serves as a histopathological hallmark of psoriasis. The previous reports demonstrated that respiratory burst, NETs, and granules from neutrophils contributed to psoriasis initiation and maintenance [50, 51]. We confirmed the pathogenic role of neutrophils in the psoriatic mice and found that MSCs-IT dramatically decreased the abundance of neutrophils in the skin, which relied on the reduced expression of CXCL1. Besides blocking neutrophil recruitment to skin lesions, MSCs-IT may also accelerate the clearance of skin neutrophils by promoting their homing to bone marrow or other locations [52]. Interestingly, the formation of NETs was also inhibited by MSCs in murine models of corneal wound healing [52, 53]. Nevertheless, the mechanisms by which MSCs exert therapeutic effects may vary greatly in different diseases.

In summary, we demonstrated a novel use of MSCs-IT in the treatment of murine psoriasis-like inflammation. TSG-6 produced by MSCs-IT can reduce neutrophil infiltration in psoriasis mouse model by decreasing the expression of CXCL1, which may be related to the reduced level of STAT1 phosphorylation. While inhibitory effect of TSG-6 on neutrophil infiltration was demonstrated in the psoriatic mice, the findings may be extrapolated to the other inflammatory disorders involving neutrophils.

## Materials and methods

### Patients and specimens

All skin tissues were obtained in agreement with the institutional guideline and the approval of the Ethics Committee of The First Affiliated Hospital of Soochow University (20220238). Informed consent was obtained from the patients that enrolled in our study. The patients with psoriasis (14 men and 16 women, age ranging from 20 to 60 years with a mean of 48.6 years old) visited the department at the First Affiliated Hospital of Soochow University without other systemic diseases and treatment.

### MSCs isolation, identification

All procedures were carried out following the rules set by Ethics Committee of Soochow University. We obtained umbilical cord and stored them in sterile phosphate-buffered saline (PBS) containing penicillin and streptomycin. To isolate MSCs, we washed the blood from the umbilical cord and removed arteries and veins. Then we minced it into small pieces, which were next transferred to 10 cm diameter dishes in Dulbecco’s modification of Eagle’s medium (DMEM) supplemented with 10% fetal bovine serum (FBS), 2 mM glutamine, 100 mg/mL penicillin and streptomycin (all from Invitrogen, CA). The medium was changed every two days. Then the cells were harvested after confluency reached 80–90%. The MSCs were identified using cell surface antigens, including CD29 (+), CD44 (+), CD73 (+), CD90 (+), CD105 (+), HLA-DR (−), CD31 (−), CD34 (−) and CD45 (−). The antibodies were purchased from Biolegend (San Diego, CA).

### Animals and IMQ-induced psoriasis mouse model

Male C57BL/6 mice, 8–10 weeks old, were purchased from Charles River Experimental Animal Technology Co.Ltd. (Beijing, China) and kept under specific pathogen-free conditions. The experiments were approved by the Institutional Animal Care and Use Committee of Soochow University (SUDA20210916A02). The mice would receive a daily topical dose of 62.5 mg IMQ cream (5%) (Aldara, 3 M Pharmaceuticals, MN) on their shaved back for six consecutive days. The mice in the control group were also shaved and applied equal doses of Vaseline. The mice were assigned to each group randomly, and the investigators were blinded to the group allocation during the experiment. Based on the scoring system called PASI, we scored erythema, scaling, and thickness on the scores from 0 to 4: none 0; slight 1; moderate 2; marked 3; very marked 4.

### Flow cytometry analysis

The lesions were removed and cut into pieces. Cell suspensions of skin were prepared after digestion with collagenase I (1 mg/mL, Invitrogen) for 2 h at 37 °C, which was achieved by pressing digested tissue with the cell strainers (70 µm). The single cell suspensions of skin and spleen were marked with the targeted antibodies, including 7-aminoactinomycin D (420403, Biolegend), anti-mouse CD45 (157605, Biolegend), anti-mouse CD3 (100203, Biolegend), anti-mouse CD11b (A15390, eBioscience, CA), anti-mouse F4/80 (25480182, eBioscience), anti-mouse Ly6g (127633, Biolegend) and anti-mouse CD11c (117307, Biolegend). All the samples were harvested by Cytoflex Flow Cytometer (Beckman Coulter, CA).

### Subcutaneous injection of MSCs

2 × 10^6^ MSCs suspended in 150 µL PBS was administered subcutaneously on the 1st day and the 4th day after IMQ application. The mice in the control group received an equal volume of PBS by subcutaneous injection.

### In vivo infusion of anti-Gr-1 mAb to deplete neutrophils

The mice were injected with 50 µg anti-mouse anti-Gr-1 or IgG (Biolegend) dissolved in 150 µL PBS intraperitoneally every other day from day −2 to 7. Skin samples on day 7 were collected for Hematoxylin-eosin staining (H&E) and flow cytometry. The results of skin samples on the 7th day showed successful depletion of Ly6G^+^ cells by anti-Gr-1.

### Quantitative real-time PCR

Total RNA of each sample was extracted by Trizol (Thermo Fisher Scientific, Waltham, MA). According to the manufacturer’s protocol, we prepared cDNA by PrimeScript RT Master Mix (Invitrogen) with 1 µg RNA template. mRNA levels were quantified by quantitative real-time PCR (qPCR) analysis using the SYBR Green Master Mix (Thermo Fisher Scientific). The primers of the targeted genes were listed in the table (Supplementary Table 1). Thermocycling included incubation at 95 °C for 20 s, followed by a program of 95 °C for 15 s and 60 °C for 15 s, for a round of 40 cycles. The amount of mRNA was compared with endogenous β-actin mRNA.

### H&E and IHC

The skin tissues of psoriatic mice were fixed in the 4% paraformaldehyde for 48 h at room temperature. Then the tissues were dehydrated through treatment with 75, 85, 95, and 100% ethanol sequentially. Next, the samples were treated with xylene before being embedded in paraffin. The samples were sectioned into 4 µm, which were analyzed by standard process.

Skin sections were stained with Ki-67 (ab15580, Abcam, Cambridge) and Ly6G antibodies (ab238132, Abcam) after deparaffinization, rehydration, and antigen repair. Then the samples were incubated with secondary antibody (Sigma) at room temperature for 1 h. The samples were stained with hematoxylin. Finally, the images were captured under a microscope (Leica, Wetzlar, Germany).

### Statistical analysis

Statistical analysis was performed by GraphPad Prism 6 software. All the data values were presented as means ± SEM. The statistical significance was assessed by student’s *t* test for the two-group comparison, and they were assessed by one-way ANOVA or two-way ANOVA for multiple-group comparison when no significance in F-test. Each experiment was repeated at least two times. *P* < 0.05 was considered statistically significant.

Supplementary information is available on (Cell Death & Disease)’s website.

## Supplementary information

Supplemental Material

Reproducibility checklist

## Data Availability

The data that support the findings of the current study are available from the corresponding author on reasonable request.
