# Supplementary material for: Mesenchymal stem/stromal cells primed by inflammatory cytokines alleviate psoriasis-like inflammation via the TSG-6-neutrophil axis
Source: Cell Death Dis. 2022 Nov 25;13(11):996. doi: 10.1038/s41419-022-05445-w (PMC9700741; doi:10.1038/s41419-022-05445-w)
Supplement: Supplementary file 1 — Supplemental Material [file 41419_2022_5445_MOESM1_ESM.docx]

**Supplementary figure 1. MSC-IT treatment does not reduce the infiltration of other immune cells.**


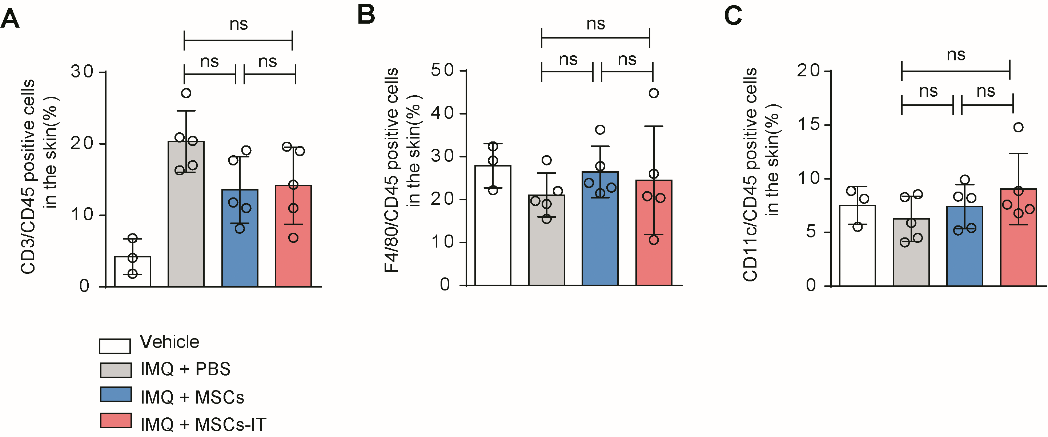


A: The CD3 positive cells (T cells) of the skin in each group were visualized by flow cytometry. B: The F4/80 positive cells (macrophages) of the skin in each group were visualized. C: The CD11c positive cells (dendric cells) of the skin in each group were visualized.

**Supplementary figure 2. The expression levels of anti-inflammatory factors.**


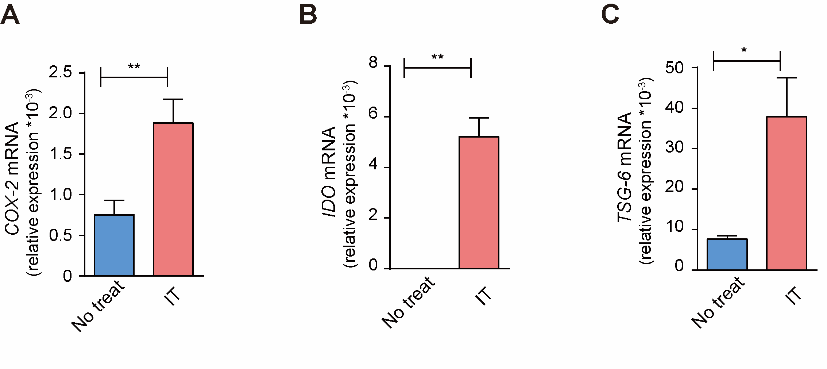


A: The relative expression of *COX2*. B The relative expression of *IDO-1*. C: The relative expression of *TSG-6*.

**Supplementary table 1. The primers were listed.**

| Primer name | Sequence（5’-3’） |
| --- | --- |
| Mouse *Cxcl1* forward primer | CCCTCTCCTTCCTCATTCTTACA |
| Mouse *Cxcl1 reverse primer* | *AGTCTTGAAAGCCCATGTGAAA* |
| Mouse *β-actin* forward primer | GTGACGTTGACATCCGTAAAGA |
| Mouse *β-actin* reverse primer | GCCGGACTCATCGTACTCC |
| Human *TSG-6* forward primer | TTTCTCTTGCTATGGGAAGACAC |
| Human *TSG-6* reverse primer | GAGCTTGTATTTGCCAGACCG |
| Human *IDO* forward primer | GCCCTTCAAGTGTTTCACCAA |
| Human *IDO* reverse primer | CCAGCCAGACAAATATATGCGA |
| Human *COX-2* forward primer | AATCCTTGCTGTTCCCACCCA |
| Human *COX-2* reverse primer | GCTTTTGTAGCCATAGTCAG |
| Human *β-ACTIN* forward primer | TTGCCGACAGGATGCAGAAGGA |
| Human *β-ACTIN* reverse primer | AGGTGGACAGCGAGGCCAGGAT |
